# On Viscous Flow in Glass-Forming Organic Liquids

**DOI:** 10.3390/molecules25174029

**Published:** 2020-09-03

**Authors:** Michael I. Ojovan

**Affiliations:** 1 Department of Materials, Imperial College London, South Kensington Campus, Exhibition Road, London SW7 2AZ, UK; m.ojovan@imperial.ac.uk or m.i.ojovan@gmail.com; Tel.: +44-747-828-9098; 2 Department of Radiochemistry, Moscow State University Named after M.V. Lomonosov, Leninskie Gory 1, Bd.3, 119991 Moscow, Russia

**Keywords:** glass-forming liquids, viscous flow, viscosity, activation energy, glass transition temperature

## Abstract

The two-exponential Sheffield equation of viscosity η(T) = A_1_·T·[1 + A_2_·exp(H_m_/RT)]·[1 + C·exp(H_d_/RT)], where A_1_, A_2_, H_m_, C, and H_m_ are material-specific constants, is used to analyze the viscous flows of two glass-forming organic materials—salol and α-phenyl-*o*-cresol. It is demonstrated that the viscosity equation can be simplified to a four-parameter version: η(T) = A·T·exp(H_m_/RT)]·[1 + C·exp(H_d_/RT)]. The Sheffield model gives a correct description of viscosity, with two exact Arrhenius-type asymptotes below and above the glass transition temperature, whereas near the T_g_ it gives practically the same results as well-known and widely used viscosity equations. It is revealed that the constants of the Sheffield equation are not universal for all temperature ranges and may need to be updated for very high temperatures, where changes occur in melt properties leading to modifications of A and H_m_ for both salol and α-phenyl-*o*-cresol.

## 1. Introduction

**Figure 1:**
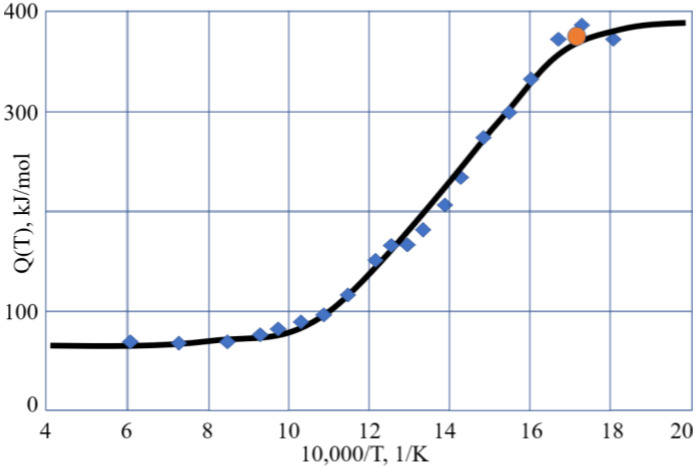
The activation energy of the viscosity Q(T) for vitreous and molten B_2_O_3_. Experimental data are taken from [23]. The orange circle indicates the position of glass transition temperature T_g_ = 580 K.

The salient feature characterizing a supercooled liquid is the dramatic increase of viscosity η(T) with decreasing temperature T, which may encompass some 15 orders of magnitude over a temperature range of almost several hundred K [1,2,3,4,5,6,7]. The interest in analyzing the viscous flow in glass-forming materials is not diminishing, with many novel findings having occurred over the last decade [8,9,10,11,12,13,14,15,16,17,18,19,20,21,22]. There are many theoretical models that can describe the viscous flow of glass-forming materials, which provide reasonably exact descriptions of viscosity–temperature relationships [1,2,3,4,5,6,7,8,9,10,11,12,13,14,15,16,17,18,19,20,21,22]. Apart from the clear physical parameters used in the models, one of important features of the models is the asymptotic description of viscosities far from the transformation range, e.g., near the glass transition temperature T_g_. It is well-known that at high and low temperatures, the viscosities of amorphous materials have an Arrhenius-type behavior η(T) = A·T·exp(Q/RT), a fact that is widely used in practice. The deviation from the Arrhenius-type behavior can be described by the activation energy of the viscous flow Q(T), dependent on temperature T. Q(T) changes from its highest value Q_H_ typical for low temperatures T < T_g_, e.g., for glasses, to its lowest value Q_L_ at high temperatures T >> T_g_, (or more exactly at exp(T/T_g_) >> 1; see the Discussion chapter), such as for melts. The typical variation of the activation energy of the flow with temperature is illustrated by Figure 1.

Stickel et al. [2] observed that the highly resolved temperature dependence of the dynamics in salol does not follow a particular function, such as the Vogel–Fulcher–Tammann (VFT) law, over the accessible range of temperatures and that none of the common routes for rationalizing the dynamics, such as Arrhenius, VFT, Souletie scaling, and idealized mode-coupling theory, account for the experimental findings properly. Nevertheless, the VFT behavior was obeyed within the limits of 265 K ≤ T ≤ 320 K, i.e., for temperatures ranging from significantly above the glass transition at T_g_ = 220 K to far above the melting point [2]. Kivelson et al. [3] attempted to assess the applicability of various competing theoretical models by examining the temperature dependence of the viscosity η(T) of a wide variety of supercooled liquids, concluding that there is a single dominant species-independent, non-molecular mechanism underlying α relaxation for all supercooled liquids throughout the entire temperature range. It was found that the overall best fits over the entire temperature range above the glass transition temperature are given by the expression T·ln[η(T)/η_∞_] = E_∞_ + BT*[(T* − T)/T*]^8/3^Θ(T* − T), where Θ(T* − T) is the Heaviside step function and T* is usually greater than the melting point temperature. More recently, the Eyring viscosity equation typically used for glass-forming liquids [12,15] was applied to calculate the viscosity, resulting in a modified temperature-dependent Eyring viscosity equation [19]. It was shown that that different regression methods exert a great effect on the final prediction results, although the viscosity of a series of glasses across a wide temperature range was accurately predicted via the optimal regression method [19]. Recent analysis revealed the general structural origin of slow dynamics in glass-forming systems, with strong local structure dynamics correlations with attractive interactions, which affect the liquid structure in a non-perturbative manner [21]. The transition from Arrhenius to non-Arrhenius viscosity behavior between Q_H_ and Q_L_ is observed in glass-forming liquids, in conjunction with anomalies in multiple thermodynamic variables, including the heat capacity, the thermal expansion coefficient, and the isothermal compressibility [22]. Moreover, it was found that the transition occurs very sharply over a temperature interval of about 15 K for salol, *o*-terphenyl, and α-picoline [11]. Doremus proposed the use of the ratio between two activation energies that are well-defined constants of materials, R_D_ = Q_H_/Q_L_, as a universal and well-defined fragility index of materials, which shows the steepness of the temperature dependence of the viscosity [5]. Short (or fragile) glass melts that have steeper temperature viscosity behavior are, therefore, characterized by high values of R_D_ > 2, whereas long (or strong) glass melts have parameter R_D_ < 2 and demonstrate a relatively weaker change of flow activation energy. One of models that incorporates such viscosity behavior is the Sheffield model, which was shown to give a very exact description of the viscosity–temperature relationships of oxide glasses within a wide temperature range, where the viscosity changes its activation energy from Q_H_ to Q_L_ [8]. It is, however, not known how well this model can be applied for organic materials that are very sensitive to temperature changes, with potential structural rearrangements that may require additional adjustments in using the equation. The objective of this paper is to demonstrate that the Sheffield model works for organics and to identify its limits when describing the viscosity dependence, using the same equation with and without parameter adjustment. 

## 2. Theoretical

The viscosity quantifies the resistance of material to flow and indicates the ability to dissipate momentum. At the microscopic level, the viscosity arises because of a transfer of momentum between fluid layers moving at different velocities. The tighter the bound layers, the more difficult their motion and the higher the resulting viscosity. As suggested by Mott [24], viscous flow occurs due to flow defects, in which the viscosity is inversely proportional to the concentration of the defects. In 1949, Ronald W. Douglas of the University of Sheffield (UK) devised a model of viscous flow based on the dual roles of oxygen in glasses, which resulted in a two-exponential equation for the temperature dependence of viscosity [25]. Although the equation gave a very good description of viscosity, it has not become popular compared with Vogel–Fulcher–Tammann (VFT), Williams–Landel–Ferry (WLF), Avramov–Milchev, Nemilov, Sanditov, Mauro–Yue–Ellison–Gupta–Allan (MYEGA), and other often used models [1,2,3,4,5,6,7,8,9,10,11,12,13,14,15,16,17,18,19,20,21,22]. It is considered that the two-exponential equations such as that obtained by Douglas can exactly describe the viscosity of amorphous materials, as the two-exponential equations with two activation energies can properly account for the asymptotic Arrhenius-type dependences of the viscosity on temperature, having different activation energies at low and high temperatures (compared with T_g_)—low Q_L_ at high and high Q_H_ at low temperatures [1]. It should, however, be noted that at very high temperatures, there are deviations from Arrhenius behavior due to critical behavior [26]. The two-exponential equation of viscosity was derived in the 2000s at the University of Sheffield using the notion of defects that assist (facilitate) flow in amorphous materials—configurons, i.e., broken chemical bonds [27,28]:

η(T) = A_1_·T·[1 + A_2_·exp(H_m_/RT)]·[1 + C·exp(H_d_/RT)] (1)

Here, A_1_ = k/6πrD_0_; k is Boltzmann constant; r is the configuron radius; T is temperature; A_2_ = exp(−S_m_/R); R = 8.314 J/mol·K is the absolute gas constant; C = exp(−S_d_/R); D_0_ = fgλ^2^zp_0_ν_0_; H_d_ and S_d_ are the enthalpy and entropy of the configuron (broken bond) formation, respectively; H_m_ and S_m_ are the enthalpy and entropy of the configuron motion, respectively; f is the correlation factor; g is a geometrical factor (~1/6); λ is the average jump length; z is the number of nearest neighbors; p_0_ is a configuration factor; ν_0_ is the configuron vibrational frequency or the frequency with which the configuron attempts to surmount the energy barrier to jump into a neighboring site. 

Comprehensive comparisons of viscosity models available for a number of oxide glasses were provided by Starodub et al. [16], Sturm [17], and Chen et al. [19]. Starodub et al. [16] demonstrated that the outcomes of modeling can be effectively improved by using machine learning techniques applied to multiparameter tasks. Chen et al. [19] found that the viscosity values simulated by using both high-temperature and low-temperature viscosity data show higher accuracy than those using only high-temperature viscosity data. Although the Sheffield model in Sturm’s analysis [17] did not rank the best among known viscosity models, it provides a direct link between the bond’s strength and the activation energy of viscosity, which reveals the processes behind the variation of activation energy. In contrast to many other approximations, Equation (1) can be used over a wider temperature range and gives the correct Arrhenius-type asymptotes at high and low temperatures, namely η(T) = A·T·exp(H_m_/RT), where A = A_1_A_2_, and η(T) = A·C·T·exp[(H_m_ + H_d_)/RT], respectively. 

The low activation energy of the flow at high temperatures is Q_L_ = H_m_, whereas the high activation energy is Q_H_ = (H_m_ + H_d_) at low temperatures. The physical meaning of these equalities is straightforward—at high temperatures, the defects of the flow in the form of broken bonds (configurons) are so abundant that the only barrier to overcome is due to configuron motion i.e. a sort of friction between imaginary layers of liquid. In contrast, at low temperatures it is necessary to create flow defects; that is, to break the bonds. Therefore, the activation energy is higher because of the enthalpy of the formation of configurons (H_d_). 

For the activation energies of the viscosity, Volf gives the following data: Q_L_ = 80–300 kJ/mol for the low viscosity range, e.g., when Log(η/Poise) < 3; Q_H_ = 400–800 kJ/mol for the high viscosity range, e.g., when Log(η/Poise) > 10 [1]. Although within the intermediate ranges of temperatures it gives practically the same description of viscosity as other commonly used models, the Sheffield equation has two asymptotic Arrhenius-type limits at high and low temperatures, with low activation energy at high temperatures and high activation energy at low temperatures. The low activation energy equals the enthalpy of motion of configurons H_m_, whereas the high activation energy equals the sum of the enthalpies of motion with the enthalpy of formation of configurons H_m_ + H_d_. Moreover, the Sheffield equation shows that all materials have a minimal achievable viscosity, which was recently confirmed by quantum mechanical analysis of Tracheko and Brazhkin [29].

Equation (1) has been used with many materials and has been proven to be valid universally at both low (for glasses) and high (for liquids) temperatures [8,30,31,32]. It is also worth noting that the first exponential term of Equation (1) is extremely high in terms of compared unity, which means that the term [1 + A_2_·exp(H_m_/RT)] in Equation (1) can be substituted for simply A_2_·exp(H_m_/RT). This reduces Equation (1) to an equation with only 4 fitting parameters (A = A_1_·A_2_, H_m_, C, and H_d_) instead of 5 fitting parameters:

η(T) = A·T·exp(H_m_/RT)·[1 + C·exp(H_d_/RT)] (2)

There is no need to use 5 fitting parameters (A_1_, A_2_, H_m_, C, and H_d_) in calculations, as Equation (2) with only 4 fitting parameters suffices for an exact description of viscosity. We should, however, account for the fact that approximation of the temperature-independent enthalpy and entropy of the formation and migration of configurons (H_d_ and S_d_, H_m_, and S_m_) is not always adequate over all temperature ranges and cannot be extended for indefinite temperatures. Those energies and entropies should depend on the material density at first, so that thermal expansion must have an effect on them (see [6,20]). Physical transformations in the materials (e.g., structural changes or boiling) can also significantly change these parameters. Therefore, we cannot always expect the same coefficients A and H_m_ at very high temperatures as those that are based on processing data from temperatures lower than the melting temperatures of materials. In this high temperature range, we should again find coefficients A and H_m_ and compare them with lower temperature data for consistency. 

The glass transition temperature in the configuron percolation theory of glass transition is given by:

T_g_ = H_d_/{S_d_ + R·ln[(1 − ϕ_c_)/ϕ_c_]} (3)

where ϕ_c_ is the percolation threshold, which determines when the first time a percolation cluster made of broken bonds—configurons—is formed [28]. Therefore, we can substitute the term C = exp(−S_d_/R) related to the entropy of the formation of configurons S_d_ in Equation (2) by C = [(1 − ϕ_c_)/ϕ_c_]·exp(−H_d_/RT_g_), which leads to:

η(T) = A·T·exp(H_m_/RT)·{1 + [(1 − ϕ_c_)/ϕ_c_]·exp[(H_d_/R)(1/T − 1/T_g_)]} (4)

Equation (4) can be further exploited, as it explicitly relates the viscosity to the glass transition temperature. The T_g_ in (4) is the temperature, which is found via differential scanning calorimetry measurements, however here it is not presumed to result in log[η(T_g_)] = 12, as typically is the case in other models (e.g., [18]).

At temperatures below the T_g_, the Sheffield equation simplifies to the following equation:

η(T) = A·[(1 − ϕ_c_)/ϕ_c_]·exp[(−H_d_/RT_g_]·T·exp[(H_m_ + H_d_)/RT)] (5)

Hence, we can find from the low temperature range the high activation energy of the flow Q_H_ = (H_m_ + H_d_) and the percolation threshold ϕ_c_, which for strong liquids is ≈0.15 [8]. At temperatures far above the T_g_ when exp(T/T_g_) > 1, the Sheffield equation simplifies into the following equation:

η(T) = A·T·exp(H_m_/RT) (6)

Hence, from the high temperature range, we can find both the constant A and the low activation energy of the flow Q_L_ = H_m_. In this range, the Sheffield equation reveals a relatively shallow minimum viscosity. This can be readily found using the Equation (6) as follows:

η_min_ = e·A·H_m_/R, (7)

where e = 2.71828 is the Euler number. The minimum viscosity occurs at the temperature found from the below equation:

T_vm_ = H_m_/R (8)

The Sheffield model of viscosity is universal for all kinds of amorphous materials and enables a good description of viscous flow in both glasses and melts. This is demonstrated in the current paper for two glass-forming organic materials—salol and cresol—also aiming to identify the limits of the model while describing the viscosity dependence with the same equation with or without parameter adjustment. Other materials with known data on temperature relationships of viscosity can also be analyzed, however this is out of the scope of this publication. 

## 3. Viscosity of Salol

Salol, i.e., phenyl salicylate C_13_H_10_O_3_, is used in the manufacture of some polymers, lacquers, adhesives, waxes, and polishes. Salol is also important for glass science as a model material (e.g., see the detailed analysis of salol dynamics in [2]). We have analyzed data on the temperature dependence of the viscosity of salol and α-phenyl-*o*-cresol taken from [33]. The coefficients of viscosity in Equations (1) and (2) were found using the best fitting procedure, utilizing both analytic [30] and genetic algorithm [8] approaches. Table 1 lists these coefficients, which are directly related to the thermodynamic parameters of configurons, e.g., enthalpies and entropies of formation and motion [8,27]. 

**Figure 2:**
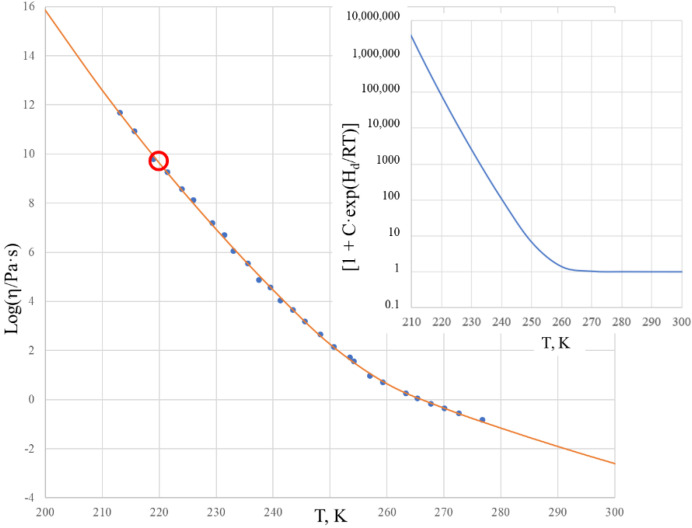
The viscosity of vitreous and molten salol. The theoretical curve was calculated using the two Sheffield equations (Equations (1) and (2)). Experimental data were taken from [33], with the red circle indicating the T_g_ = 220 K [33]. The inset shows the temperature dependence of factor [1 + C·exp(H_d_/RT)] in Equations (1) and (2).

Figure 2 shows the viscosity of salol within the temperature range of 200–300 K in a logarithmic scale. The solid curve is calculated by the two Sheffield equations (Equations (1) and (2)) using the parameters from Table 1. 

Comparison of the experimental data with those calculated using the Sheffield equation reveals a very good description of the viscosity, with the root mean squared error (RMSE) characterizing the fit (RMSE = 0.085). We note that the glass transition temperature of salol is T_g_ = 220 K, as found from the heat capacity dependence on temperature [33], and we observe that the viscosity of salol at the glass transition temperature Log[η(T_g_)] = 9.64 is significantly (hundreds of times) below the generic and arbitrary definition of the glass transition as taking place at Log[η(T_g_)] = 12 [34,35,36]. There is a clear change of the activation energy of the viscous flow from its high value Q_H_ = 263.6 kJ/mol at temperatures below ~220 K to its low value Q_L_ = 118.4 kJ/mol at temperatures above ~260 K. The temperature T_2_ = 260 K, above which the viscosity of salol is described by the Arrhenius-type equation (6), is revealed by the inset of Figure 2, which shows that the term [1 + C·exp(H_d_/RT)] is practically equal to 1 above 260 K, and only below T_2_ shall it be accounted for as deviating from the unity. Figure 2 also shows that above temperature T_2_ the Arrhenius behavior of the viscosity is not much different to T_g_. This means that all temperatures above T_g_ can be used in finding the fitting coefficients of the Sheffield equation by applying the analytical procedure described in [30].

It is also worth noting that the first exponential term of the Sheffield equation (Equation (1)) is extremely high compared with unity, e.g., it changes from 9.6·10^28^ at T = 200 K to 2.7·10^10^ at T = 500 K. This confirms that Equation (2) can be used instead of Equation (1) without any loss of accuracy. There is no need to use 5 fitting parameters in the calculations, as the Equation (2) with only 4 fitting parameters suffices for an exact description of the viscosity. Finally, we can calculate the Doremus fragility ratio R_D_ = Q_H_/Q_L_ using the data from Table 1 as follows: R_D_ = 1 + H_d_/H_m_. One can see that salol is a typical fragile liquid with a Doremus fragility ratio of R_D_ = 2.2, which is not much above 2. 

**Figure 3:**
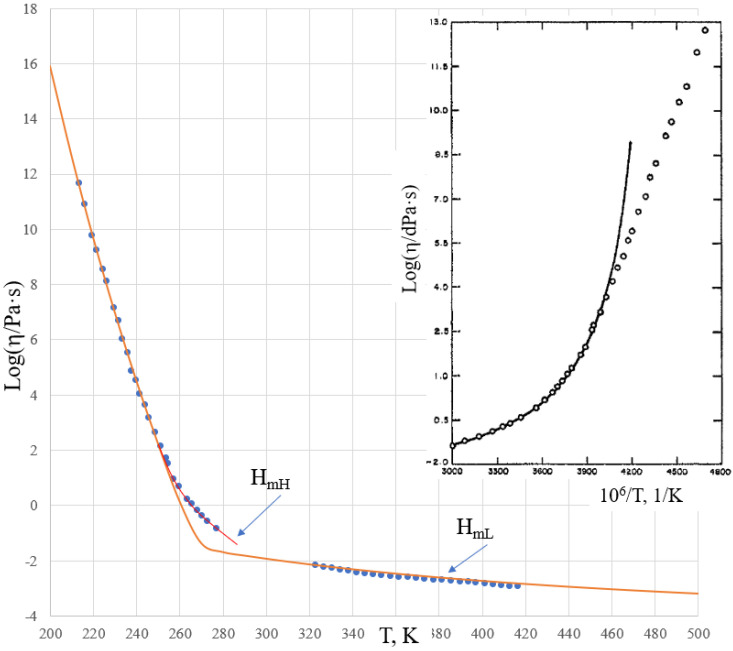
The viscosity of vitreous and molten salol over a wide temperature range. The theoretical curve was calculated using Equation (2), with the same parameters A, H_m_, C, and H_d_ used at all temperatures. Experimental data were taken from [33] for lower temperatures (T < 280 K) and from [37] for the high temperature range (T > 320 K). The inset shows the viscosity curve by VFT approximation [33].

The viscosity of salol was further analyzed at high temperatures by Cukierman, Lane, and Uhlmann [37]. These data revealed that the activation energy of the flow, e.g., H_m_ in Equation (6), is much lower than that obtained above (Table 1). Using high-temperature viscosity data for salol from [37], an attempt was made to use the Sheffield equation throughout all temperature ranges using a modified set of fitting parameters, which is shown in Figure 3.

Deviations of the theoretical curve in the experiment within the temperature range of 260–280 K are unacceptably large. We recall that the behavior in this range is well described by Equation (2) when using data from [33] only (see Figure 2). This reveals that parameters A and H_m_ in the high temperature range differ significantly from those obtained using data closer to T_g_, as in given in Table 1. This demonstrates that the Sheffield equation, although describing the trends for the temperature dependence of the viscosity well, fails to exactly describe these trends based on the same parameters throughout all temperature ranges. Deviations from the experiment, as seen in Figure 3, are unacceptable in the range where the viscosity is already described by the Arrhenius-type relationship (6), although the parameters of this relationship can change due to thermal expansion, structural rearrangements, boiling, etc. The enthalpy of the motion of configurons H_m_ dropped from H_mL_ = 118.41 kJ/mol at temperatures below 280 K (see Table 1) to H_mH_ = 21.3 kJ/mol above 320 K, which most probably was due to the structural complexity of salol molecules, as shown by the inset in Figure 3. 

## 4. Viscosity of Cresol

Cresol, i.e., α-phenyl-*o*-cresol, belongs to the group of organic compounds of cresols that are precursors to many compound materials, including plastics, pesticides, pharmaceuticals, and dyes. We have analyzed data on the temperature dependence of the viscosity of α-phenyl-*o*-cresol taken from [24]. The viscosity coefficients in Equations (1) and (2) were found using the best fitting procedure, utilizing both analytic [30] and genetic algorithm [8] approaches (Table 2). 

**Figure 4:**
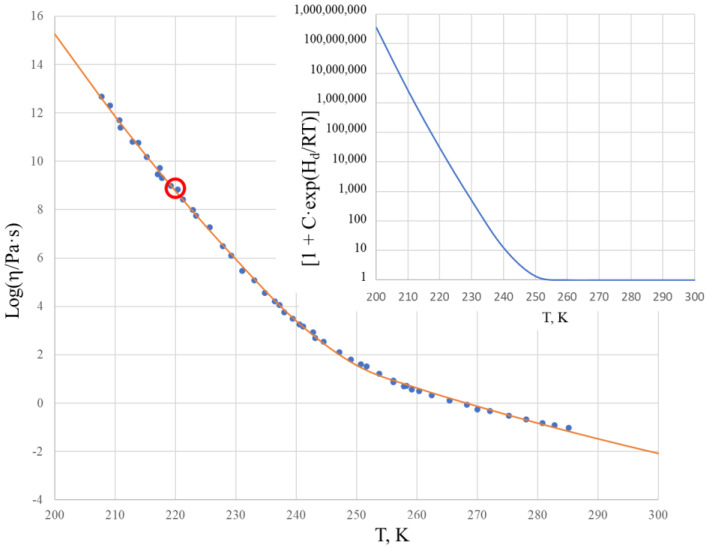
The viscosity of vitreous and molten α-phenyl-*o*-cresol using the two Sheffield equations (Equations (1) and (2)). Experimental data were taken from [33], with the red circle indicating T_g_ = 220 K [33]. The inset shows the temperature dependence of factor [1 + C·exp(H_d_/RT)] in Equations (1) and (2).

Figure 4 shows the viscosity of α-phenyl-*o*-cresol within the temperature range of 200–300 K in a logarithmic scale, calculated using the two Sheffield equations (Equations (1) and (2)) with experimental data taken from [33]. 

Comparison of the experimental data with the calculated data reveals the very good description of the viscosity using the Sheffield equation, with RMSE = 0.11. With the glass transition temperature T_g_ = 220 K [33], the α-phenyl-*o*-cresol has a logarithm of viscosity at T_g_ as high as 8.78, which means that the viscosity is more than 1500 (1659) times below the generically considered value Log[η(T_g_)] = 12 [34,35,36]. The viscosity shows a significant change of activation energy from its high value Q_H_ = 275.37 kJ/mol at temperatures below ~220 K to its low value Q_L_ = 103.22 kJ/mol at temperatures above T_2_ = 240 K, where the viscosity can be described by the Arrhenius-type equation (Equation (6)). The inset of Figure 4 shows that the term [1 + C·exp(H_d_/RT)] is practically equal to 1 above 240 K, and only below this shall it be considered to deviate from the unit. Similarly to salol, the first exponential term of the Sheffield equation for α-phenyl-*o*-cresol is extremely high, i.e., it changes from 1.22·10^26^ at T = 200 K to 8.14·10^9^ at T = 500 K, meaning that the term [1 + A_2_·exp(H_m_/RT)] in Equation (1) can be substituted for A_2_·exp(H_m_/RT), which reduces the first Sheffield equation (Equation (1)) to its simplified form (Equation (2)) with only 4 fitting parameters, e.g., A, H_m_, C, and H_d_. The Doremus fragility ratio is R_D_ = 2.67, demonstrating that α-phenyl-*o*-cresol is a more fragile liquid compared with salol.

We note that similarly to salol, attempts have failed to model the viscosity–temperature relationships of cresol when accounting for the high temperature range data from [38] and using the same parameters A, H_m_, C, and H_d_ in Equation (2) at all temperatures. The enthalpy of motion of the configurons of α-phenyl-*o*-cresol H_m_ dropped from H_mL_ = 103.22 kJ/mol for temperatures below 290 K (Table 2) to H_mL_ = 25.62 kJ/mol above 320 K. This reveals that similarly to salol, the Sheffield equation cannot be used for α-phenyl-*o*-cresol with the same thermodynamic constants H_d_, S_d_, H_m_, and S_m_ at all temperatures. 

## 5. Discussion

The temperature dependence of viscosity of amorphous materials η(T) is a continuous function of the temperature T, which has two exact Arrhenius-type asymptotes at high and low temperatures compared with T_g_. At intermediate temperatures, the activation energy of the viscous flow Q(T) is a function of the temperature, e.g., it can be used in an Arrhenius-type equation η(T) = A·T·exp(Q/RT), where it formally depends on the temperature. There are many effective models of viscosity to account for this [1,2,3,4,5,6,7,8,9,10,11,12,13,14,15,16,17,18,19,20,21,22,39,40,41,42,43], with two of the most frequently used models being the Williams–Landel–Ferry (WLF) equation for polymers and the Vogel–Fulcher–Tammann (VFT) equation for inorganic materials. The WLF equation typically used for polymers is [39]:

η(T) = η_0_·exp[−C_1_·(T − T_0_)/(C_2_ + T − T_0_)] (9)

where η_0_ is a constant and T_0_ is taken as T_g_, whereas C_1_ and C_2_ are universal constants for most polymeric materials. The VFT equation of viscosity describes viscosity data at intermediate temperatures over many orders of magnitude with quite high accuracy [7]:

η(T) = η_0_·exp[B/(T − T_0_)] (10)

where η_0_, B, and T_0_ (Vogel temperature) are material specific constants. Although both (9) and (10) give very good descriptions of viscosity within an intermediate range of temperatures, neither correctly describe the asymptotic behavior of viscosity, which is naturally of the Arrhenius-type. As noted in [33], the VFT equation greatly overestimates the viscosity for both salol (see the inset of Figure 3) and cresol. 

The above results for the temperature behavior of the viscosity of salol and cresol have shown that the four-parameter equation (Equation (2)) can be used with practically the same results as the Sheffield equation (Equation (1)), facilitating the fitting procedures needed to find parameters A, H_m_, C, and H_d_ of this equation. We note that Equation (2) is the one that Volf claimed to be the best at mathematically describing the viscosities of condensed materials [1], and it is the same type of equation that Douglass derived for silicate systems in 1949 [25]. Numerical data show that just below T_g_, the activation energy of the flow for both salol and cresol becomes constant and high, e.g., Q_H_. When exceeding the T_g_ by about 20 to 40 K, the activation energy of the flow for both salol and cresol rapidly becomes almost constant and low, e.g., Q_L_. The transformation range where the activation energy of flow Q(T) is temperature-dependent and changes from Q_H_ to Q_L_ is, thus, narrow in the temperature scale, although the changes in the viscosity are drastic and cover many orders of magnitude. We also observe that the actual changes of Q(T) occur only above T_g_ in the undercooled liquid, while in the glassy state the Q(T) is almost unchanged and equal Q_H_. As seen from Figure 1, this is also the case for B_2_O_3_, which is also a fragile liquid, with R_D_ = 3.28 [8,30]. Analysis of the activation energy for the viscosity of silica, which is a strong (long) liquid, shows that Q(T) changes over a wider range within the glassy state [43], although this range is narrow compared to T_g_. Accounting for this, we can conclude that the Arrhenius-type behavior of the viscosity with low activation energy Q_L_ occurs when exp(T/T_g_) > 1 rather than requesting T/T_g_ >> 1, which is a very strong requirement when processing experimental data [30]. This result simplifies the utilization of the analytical procedures used to determine parameters A, H_m_, C, and H_d_ of the second Sheffield equation (Equation (2)). Both Equations (1) and (2) (as seen from Figure 2 and Figure 4) resulted in very good descriptions of the viscosity within the transformation range where the activation energy varies, similar to the results given by the well-known WLF equation (Equation (9)) and VFT equation (Equation (10)). Compared with those equations, however, we can extend the temperature ranges for both low and high temperatures. This extension is not indefinite in practice, as we found above for both salol and cresol. In the high temperature range above 320 K, the enthalpy of motion for configurons for both salol and α-phenyl-*o*-cresol decreases significantly, which could be due to the relative complex molecular structures of these organic materials. Indeed, due to thermal expansion, more free volume becomes available for molecules to move [20,27]. The enthalpy of motion of configurons is analogous to the elastic strain energy in glass, with H_m_ estimated as H_m_ = πμ·(r – r_dr_)^2^·λ, where μ is the shear modulus of the glass, r is the radius of configuron and λ is average jump length as in Equation (1), and r_dr_ is the radius of the network doorway [27]. At higher temperatures, r_dr_ increases due to thermal expansion, thus diminishing H_m_, which is in line with Frenkel’s original idea that the thermal fluctuations increase the cage radius and enable the atom to escape the cage. It is notable that for simpler inorganic (oxide) systems such as diopside, using the same thermodynamic constants H_d_, S_d_, H_m_, and S_m_ the Sheffield equation gives an exact description of the viscosity–temperature relationships at all temperatures, including in the high temperature range (see Figure 1 in [8]). Therefore, the modification of H_m_ at high temperatures is material-specific and cannot be generic for all materials. 

Recently, Trachenko and Brazhkin found that all materials have a certain minimum achievable viscosity [29]. It is obvious that the Sheffield equation also exhibits a low minimum viscosity at very high temperatures, after which the viscosity increases with the increase of temperature. Based on data in the high temperature range from [37] from Equations (7) and (8), we can find both the minimum viscosities and temperatures at which these are attained (Table 3).

However, as pointed out by Louzguine-Luzgin et al. [26], the temperature dependence of the viscosity has physical limitations on the high temperature part, where in practice it is valid up to the boiling point at pressure ranges from the triple point to the critical pressure and nearly up to the critical temperature at high pressure. The temperatures where minima can theoretically be achieved by salol (2562 K) and α-phenyl-*o*-cresol (3924 K) are extremely high, which was not the case in experiment. Therefore, extension of the viscosity plots to such extremely high temperatures for these organic materials is not possible. One can expect decomposition of these molecules much before these extremely high temperatures, so minima in both materials should appear at a much lower temperature than predicted in Table 3. 

We have observed that the main variations of the activation energy of the viscous flow in salol and α-phenyl-*o*-cresol occur above T_g_. The viscosities at the glass transition temperatures of both salol (Log[η(T_g_)] = 9.64) and α-phenyl-*o*-cresol (Log[η(T_g_)] = 8.78) are several orders of magnitude below the generically accepted value of the logarithm of viscosity, which is as high as 12 at the conventional glass transition temperature [34,35,36]. Moreover, the former arbitrary definition of the glass transition temperature is often related to other viscosities; for example, Laughlin and Uhlmann used the temperature corresponding to a viscosity of 10^15^ P (e.g., 10^14^ Pa·s) as T_g_ [33]. Mazurin noted that the widespread idea that the glass transition temperatures of all glasses corresponds to the temperature at which the glass viscosity is 10^12^ Pa·s is not justified, although most of the glasses do obey this rule [44]. These data, along with data on the viscosities of other materials at different T_g_ values [45], question the concept of the universality of defining T_g_ values from the equation Log[η(T_g_)] = 12. 

The transition of glass is most often considered to be a gradual change from ergodic to non-ergodic states without structural changes, where it is assumed that the glassy state of the amorphous materials is inherently non-ergodic [46]. The observed glass transition viscosity levels below 10^12^ Pa·s for some materials, along with evident changes in the heat capacity and thermal expansion coefficient at T_g_, indicate that glass transition is a phase transformation [28,45,47,48]. This transformation is similar to a second-order phase transition in the Ehrenfest sense, with continuity of the volume and entropy and discontinuity of their derivatives, which are used in practice to identify T_g_. The glass transition is accompanied by significant structural changes, which are revealed via X-ray diffraction [49], high-precision measurements of third- and fifth-order non-linear dielectric susceptibilities that strongly support theories based on the thermodynamic amorphous order, which is fractal in its dimensions [50], as well as direct visualization of macroscopic percolating clusters formed by molecules at the glass transition [51]. The glass transition should be considered as an example of critical phenomena generically termed topological phase transitions, which are amenable to the scaling approach and characterized by diverging length and time at the transition [28,52]. Angell’s concept of configurons in covalently bonded systems [53] and Egami’s ideas of local connectivity in amorphous metals [54] allows the glass–liquid transition to be treated as a percolation-type phase transformation of the system of chemical bonds. The structural difference between glasses and liquids near T_g_ becomes obvious in terms of the Hausdorff dimensionality D of the system of configurons, so that in glasses the set of configurons has D = 0 because broken bonds are point-type defects, whereas in liquids the configurons form extended structures—macroscopic percolation clusters with the fractal dimension D = 2.5 [55]. As for the entropy, energy. and enthalpy of glasses, as emphasized by Nemilov [39,40,56], these are functionals in the thermodynamics of the vitreous state, with additional internal (structural, ordering) parameters that are used along with temperature and pressure, which determine the state of the system in Gibbs thermodynamics. The processing of experimental viscosity data is important in order to identify not only the rheological properties of materials, but also other material parameters. The Sheffield equation of viscosity provides data on chemical bond parameters, including both enthalpies and entropies. Recently developed artificial intelligence and machine learning techniques (see [16,19,57]) can effectively give detailed information on the bonding system of materials, thereby contributing to efforts to improve the properties and functionalities of novel glasses.

## 6. Conclusions

Utilization of the Sheffield equation of viscosity for glass-forming organic materials is successfully demonstrated for two cases—salol and α-phenyl-*o*-cresol. In both cases, it is numerically confirmed that the simplified variant of the Sheffield equation η(T) = A·T·exp(H_m_/RT)·[1 + C·exp(H_d_/RT)], which has 4 fitting parameters, provides data in good agreement with the experiment. It was revealed that above the glass transition temperature, when exp(T/T_g_,) > 1, and just below the T_g_ the viscosity can be described using the asymptotic versions of the Sheffield equation, which are Arrhenius-type and have low and high flow activation energies, e.g., Q_L_ = H_m_ and Q_H_ = (H_m_ + H_d_), respectively. Calculations show that the main changes of the flow activation energy in both salol and α-phenyl-*o*-cresol occur above T_g_, with variations occurring in a relatively narrow temperature range of about 20 to 40 degrees. Analysis of the viscosity behavior revealed that the parameters of the Sheffield equation are not universal and are modified in the high temperature range both for salol and α-phenyl-*o*-cresol, where the enthalpy of configuron migration H_m_ drops significantly compared with the lower temperature range. Analysis has also revealed that at the glass transition temperature, the viscosities of both salol and α-phenyl-*o*-cresol are many orders of magnitude lower than the generically used value of 10^12^ Pa·s. 

## Figures and Tables

**Table 1:** Parameters of the Sheffield equation of viscosity ^1^ for salol.

A_1_, Pa·s/K	A_2_	A = A_1_·A_2_, Pa·s/K	H_m_, kJ/mol	C	H_d_, kJ/mol
1.78·10^−24^	0.0114	2.03·10^−26^	118.41	2.57·10^−30^	145.17

^1^ Pa·s = 10 P (Poise, the non-system unit of viscosity).

**Table 2:** Parameters of the Sheffield equation of viscosity for α-phenyl-*o*-cresol.

A_1_, Pa·s/K	A_2_	A = A_1_·A_2_, Pa·s/K	H_m_, kJ/mol	C	H_d_, kJ/mol
2.2·10^−22^	0.1341	2.95·10^−23^	103.22	3.85·10^−37^	172.15

**Table 3:** Minimal theoretical viscosities.

Material	T_vm_, K	η_min_, Pa·s
Salol	2562	5.35·10^−5^
α-phenyl-*o*-cresol	3924	4.75·10^−5^
